# Eutopic/ectopic endometrial apoptosis initiated by bilateral uterine artery occlusion: A new therapeutic mechanism for uterus-sparing surgery in adenomyosis

**DOI:** 10.1371/journal.pone.0175511

**Published:** 2017-04-13

**Authors:** Li Chen, Caixia Li, Jing Guo, Ning Luo, Xiaoyan Qu, Le Kang, Mingmin Liu, Zhongping Cheng

**Affiliations:** 1 Department of Obstetrics and Gynaecology, Yangpu Hospital, Tongji University School of Medicine, Shanghai, PR China; 2 Institute of Gynaecologic Minimally Invasive Medicine, Tongji University School of Medicine, Shanghai, PR China; Duke University School of Medicine, UNITED STATES

## Abstract

The objective of the present study was to investigate differences in the expression of apoptosis-related factors in the eutopic and ectopic endometrium (EuE/EE) in women with adenomyosis before and after laparoscopic bilateral uterine artery occlusion (LUAO). Ten patients with uterine adenomyosis who received LUAO were selected as the research subjects, from whom EuE and EE tissues were obtained before and after LUAO and detected for the expression of apoptosis-related molecules in EuE and EE by PT-PCR and Western blot, and changes in the mitochondrial structure by electron microscopy. Normal endometrial stromal cells (NESC), and EuE/EE stromal cells in women with adenomyosis were cultured in a 1% O2, 5% CO2 incubator to establish a physical anoxia state in an in vitro stromal cell model. The expression of apoptosis-related molecules was observed at 0, 6, 12, 24 and 48h of hypoxic. The results showed that the expression of apoptosis-related factors in EuE and EE were increased significantly after LUAO and under hypoxic conditions in vitro, suggesting that transient ischemia and hypoxia were involved in the apoptosis of adenomysis lesions, and that uterine artery occlusion could remove adenomyosis lesions on tissue/cell level by cytoreduction, thus reaching the goal of treating adenomyosis effectively.

## Introduction

Adenomyosis is a common chronic disease primarily diagnosed in childbearing females. It is defined as the presence of endometrial glands and stroma causing reactive hyperplastic or hypertrophic myometrium., surrounded by chronic inflammation in the endometrium [1,2,3,4]. Uterus-sparing surgery is the current trend in the treatment of uterine adenomyosis to enable women to preserve future fertility and avoid the impact of a hysterectomy on sexual function and mental discomfort. Several new methods and techniques have been tentatively used in treatment of adenomyosis, including uterine artery embolism (UAE), high frequency ultrasound (HIFU), balloon endometrial thermoablation and hysteroscopic endometrial resection [5]. A study by Nijenhuis etal[6] reported that UAE using polyzene F-coated hydrogel microspheres showed good clinical outcomes in 28 (97%) of their 29 patients with treatment-resistant adenomyosis, and hysterectomy was required in only one patient. UAE was used as a potential therapy for adenomyosis in all related publications from 1999 through 2010. Long-term data are available from 511 affected females from 15 studies, with an improvements rate of 75.7% (378/511) during a median follow-up period of 26.9 months [7]. Based on the UAE method, researchers have begun performing laparoscopic uterine artery occlusion (LUAO) for uterine fibroids with satisfactory outcomes [8,9,10,11]. From 2003 to 2005, we utilized LUAO combined with partial resection for the treatment of 182 eligible patients with symptomatic adenomyosis [12]. The result showed that the postoperative menstrual quantity was decreased significantly and the uterus volume was reduced by 58.3% in these patients during the 36-months follow-up period. Their health-related quality of life was also improved significantly as compared with that before treatment. Postoperative recurrence occurred in only three (1.7%) patients, for which hysterectomy was required. Our preliminary clinical practices have demonstrated that LUAO combined with partial resection for the treatment of adenomyosis is safe and effective, and the overall outcome is superior to that reported in the literature[13]. This technology has been incorporated into the uterus adenomyosis classification treatment as an independent operation [5].

Based on a series of LUAO treatments of uterine fibroids, we originally propose the hypothesis of ‘Single organ uterus shock’ in our previous study, which may ideally explain the UAO mechanism in theory [14]. After LUAO, the uterus and myomas underwent ischaemia and hypoxia. The uterus survived due to restoration of its blood supply, but the myomas ‘died’ due to lack of blood supply and the duration of hypoxia. During the process, the uterus underwent pathophysiological changes of hypoxia-reperfusion similar to the course of shock [15]. Our previous study showed that LUAO as one kind treatment of adenomyosis achieved satisfactory clinical efficacy. Nevertheless, the exact mechanism underlying LUAO remains to be further elucidated. In this study, we observed an apoptosis phenomenon in the eutopic endometrium(EuE) and ectopic endometrium(EE) in patients with adenomyosis after LUAO, and then established a hypoxia cell models to validate this phenomenon.

## Materials and methods

### Reagents

Antibodies for Apaf-1, CHOP, TRADD, Bcl-2 and Bax were purchased from Santa (Dallas, USA). Antibodies for Endo-G, Cyt-c, AIF, GRP78, Caspase3, Caspase4, Caspase8 and Caspase-9 were purchased from Abcam (Cambridge, USA). Antibodies for glyceraldehyde-3-phosphate dehydrogenase (GAPDH) were purchased from Cell Signaling Technologies (Danvers, USA). Secondary antibodies of goat anti-mouse FITC, goat anti-rabbit HRP and goat anti-mouse HRP were purchased from the Beyotime Institute of Technology(Shanghai, China).

### Subjects

Ten patients with uterine adenomyosis who received LUAO combined with partial resection of adenomyosis were recruited in this study. The preoperative diagnosis of adenomyosis patients based on typical clinical symptoms of dysmenorrhea and/or menorrhagia and pelvic pain, physical examination findings, and imaging results including transvaginal ultrasound and MRI. The ages of the patients ranged from 34 to 45 years with a mean of 39.67±5.01 years. All control women and adenomyotic women had regular menstrual cycles (28–35 days). The exclusion criteria were patients who were diagnosed with uterine fibroid and subsequently received hormonal therapy within the past 6 months; patients with reproduction tract infections or immune system and endocrine diseases; and patients whose preoperative hysteroscopy excluded the diagnosis of endometrial lesions. The phase of the menstrual cycle was the early proliferative phase. Fresh EuE and EE tissues before and after LUAO were collected, and EuE and EE tissues obtained before LUAO were used as control. Part of them were stored at 4°C in phosphate-buffered saline (PBS) (100 U/mL penicillin, 100 μg/mL streptomycin added) and processed within 2–18 h, and the remaining part were stored at -80°C for RNA and protein extraction. In hypoxia cell models experiment, normal cell control was from patients who voluntarily required to place intrauterine device (IUD) excluding other diseases, without the use of hormone drugs nearly 3 months. Samples of endometrium obtained by pipelle or curettage before place the IUD were cultured. Details of the LUAO procedure and the sampling time were recorded. All cases included in this study were confirmed by postoperative pathology.

### Ethics statement

The study was approved by the Ethics Committee of Yang-Pu Hospital, Tongji University School of Medicine (Shanghai, China; Registration No.: LL-2014-WSJ-006), and in accordance with the tenets and guidelines of the Declaration of Helsinki. All patients provided written informed consent.

### Electron microscopy reveals histomorphological changes of EuE and EE after LUAO

EuE and EE samples were fixed in glutaric acid and osmic acid, dehydrated with pyruvic acid, epoxy resin embedding, sliced into ultra-thin sections, stained with uranyl and lead citrate, and finally observed under an electron microscope for mitochondrial morphological changes and cell apoptosis such as vague or disappearing mitochondria cristae, mitochondrial swelling or cavity changes.

### Real-time polymerase chain reaction(PCR)

Liquid nitrogen-preserved tissues were homogenized, and total RNA was extracted using the TRIzol method (Invitrogen, CA, USA). A reverse transcription kit was used to prepare cDNA. The following PCR primer sets were used in this study: Apaf-1, forward primer: 5' TCTACCTCTGCTGACAAG 3', reverse primer: 5' CACCGTTTGAGACATTCC 3'; CHOP, forward primer: 5' CAGGAAACGGAAACAGAG 3', reverse primer: 5' CACCATTCGGTCAATCAG 3'; TRADD, forward primer: 5' CCAGCCCTTACAGTTTCAC 3', reverse primer: 5' GGCAGGCAAGATTGATTCC 3'; Bcl-2, forward primer: 5' AGACCGAAGTCCGCAGAACC 3', reverse primer: 5' GAGACCACACTGCCCTGTTG 3'; Bax, forward primer: 5' AGCTGAGCGAGTGTCTCAAG 3', reverse primer: 5' TGTCCAGCCCATGATGGTTC 3'; Endo-G, forward primer: 5' TGTCTGCACAGGGCCACTCTTC 3', reverse primer: 5' TGCCTCCAGGATCAGCACCTTG 3'; Cyt-c, forward primer: 5' CTTTGTGCTGGAAGGAACC 3', reverse primer: 5' CAGGGCATTACACGCTAAC 3'; AIF, forward primer: 5' GCTACAAGCACGCTCTAAC 3', reverse primer: 5' GCCCAAATCACTCCAGAAC 3'; GRP78, forward primer: 5' CCCGTCCAGAAAGTGTTG 3', reverse primer: 5' CAGCACCATACGCTACAG 3'; Caspase3, forward primer: 5'GTTTGAGCCTGAGCAGAGAC 3', reverse primer: 5' TGGCAGCATCATCCACAC 3'; Caspase4, forward primer: 5' AATGGAGCTGACTTTGAC 3', reverse primer: 5' ATAAAGCAGCACATCTGG 3'; Caspase8, forward primer: 5' CTGGGAGAAGGAAAGTTG 3', reverse primer: 5' TTGGAGAGTCCGAGATTG 3'; Caspase-9, forward primer: 5'GGGTCGCTAATGCTGTTTC 3', reverse primer: 5' TGCTAAGAGCCTGTCTGTC 3'; GAPDH, forward primer: 5' CACCCACTCCTCCACCTTTG 3', reverse primer: 5' CCACCACCCTGTTGCTGTAG 3'. The SYBR Green kit was used, and GADPH served as an internal control. The mRNA expression level of the apoptosis-related genes Apaf-1, CHOP, TRADD, Bcl-2, Bax, Endo-G, Cyt-c, AIF, GRP78, Caspase3, Caspase4, Caspase8 and Caspase-9 in EuE and EE tissues following LUAO was detected by real-time PCR. The 2-ΔΔCT method was used to calculate the mRNA levels [16].

### Western blotting analyses

Western blotting analyses was performed to detect protein expression. Primary antibodies were incubated overnight with the appropriate primary antibody and secondary antibody. Finally, the optical density of protein signal strength was determined, using GAPDH as an internal standard to determine the protein level. The primary antibodies included anti-rabbit Bcl2 (PBST 1: 200, Santa Cruz Biotechnologies), anti-rabbit Bax (1: 200, Santa Cruz Biotechnologies), anti-rabbit cyt-c (1: 1000, Cell Signaling Technology), anti-rabbit AIF (1: 1000, Abcam), anti-rabbit caspase3 (1: 1000, CST), anti-rabbit caspase 4 (1: 1000, Abcam), anti-rabbit Caspase 8 (1:2000, Abcam), anti-rabbit Caspase 9 (1: 1000, Abcam), anti-rabbit EndoG (1: 2000, Abcam), anti-rabbit Apf1 (1: 1000, Abcam), anti-rabbit GRP78 (1: 1000, Abcam, Cambridge, MA), anti-rabbit CHOP (1: 1000, CST), anti-rabbit TRADD (1:500, Abcam), and anti-rat GAPDH (1: 1500, CST).

### Primary cell culture

Three samples of fresh EE in Hank’s Balanced Salt Solution (HBSS) containing double resistance (penicillin: 1000 U/ml, streptomycin: 1000 μg/ml) were obtained and rinsed 2–3 times. Next, 8 ml 0.4% collagenase (containing DNase I: 20 μg/ml) and 2 ml 0.05% pancreatic enzyme were added to the samples and digested at 37°C with shaking for 2–3 hours. Then, the tissues were cultured in DMEM-F12 medium (10% FBS, 100 U/ml penicillin and 100 μg/ml streptomycin) in a 37°C incubator. According to the adherent time differences of the stromal and epithelial cells, we obtained stromal cells. According to the identification of the cell morphology, stromal cells exhibited flat, spindle-shaped, fibroblastic-like morphology, and interstitial cell surface marker (vimentin) of the separated stromal cells, we established a separate cell culture system. Using this preparation method, three groups (EuE, EE and NE) were established[17]. Three patients with early proliferative phase of the menstrual cycle were used as the control group, from whom a small amount of endometrial tissue was obtained before the intrauterine device was placed.

### Construction of the hypoxia mode

An in vitro stromal cell model was established, and then the experiment cells, including normal endometrial stromal cells (NESC), EuE stromal cells (EuESC) and EE stromal cells (EESC) were cultured in a 1% O_2_, 5% CO_2_ incubator to establish a physical anoxia state, and a normal oxygen (21% O2) condition for 0, 6, 12, 24 and 48h. Next, the cells were collected, and Western blot was performed to identify the protein expression of the main apoptosis-related factors Bcl-2, Bax, caspase 3, Endo-G, caspase 8 and GRP78. The obtained results were compared between the groups.

### Statistical analysis

Using GraphPad Prism 4 (GraphPad software), data were expressed as the mean ± SD. Tissue samples were analysed using the paired t-test, and cell specimens were analysed using an independent t -test. P<0.05 was considered statistically significant.

## Results

### Electron microscopic observation of changes in EuE and EE before and after LUAO

**Fig 1:**
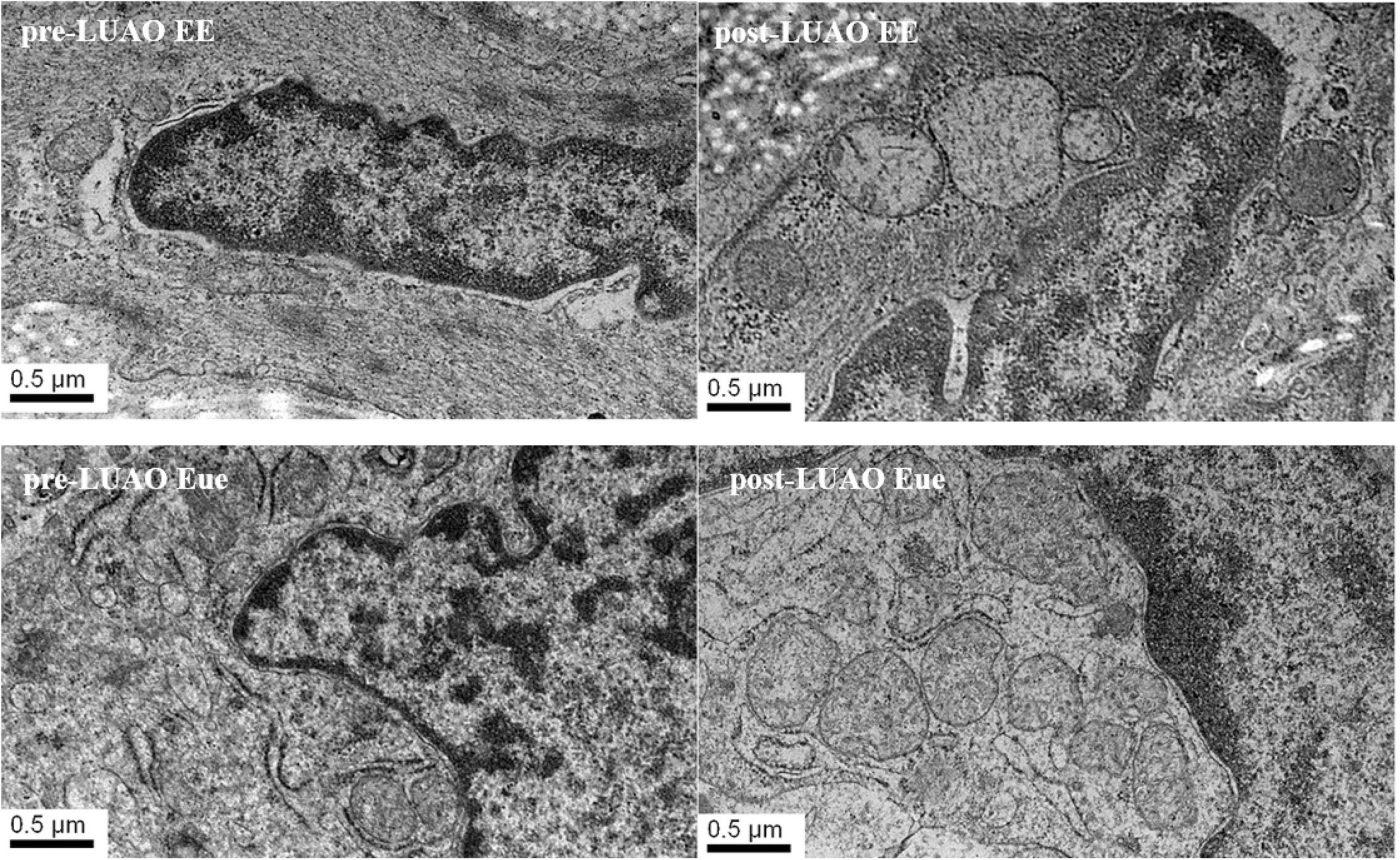
Transmission electron microscop: changes of EuE and EE before and after LUAO (30000×). Apoptosis changes mainly include swollen mitochondria, cavitation sample change, nuclear chromatin edge, cell membrane reflex, cytoplasmic concentration, and the formation of apoptotic bodies.

Electron microscopy (30000×) revealed that EuE and EE tissue in adenomyosis underwent apoptosis after LUAO. The mitochondria cristae looked vague or disappeared with mitochondria swelling and a cavitation change. In addition, there was a nuclear chromatin edge and cell membrane reflex. The cytoplasmic concentration and the formation of apoptotic bodies were observed (Fig 1).

### The mRNA expression of apoptosis-related factors in EuE and EE before and after LUAO

**Fig 2:**
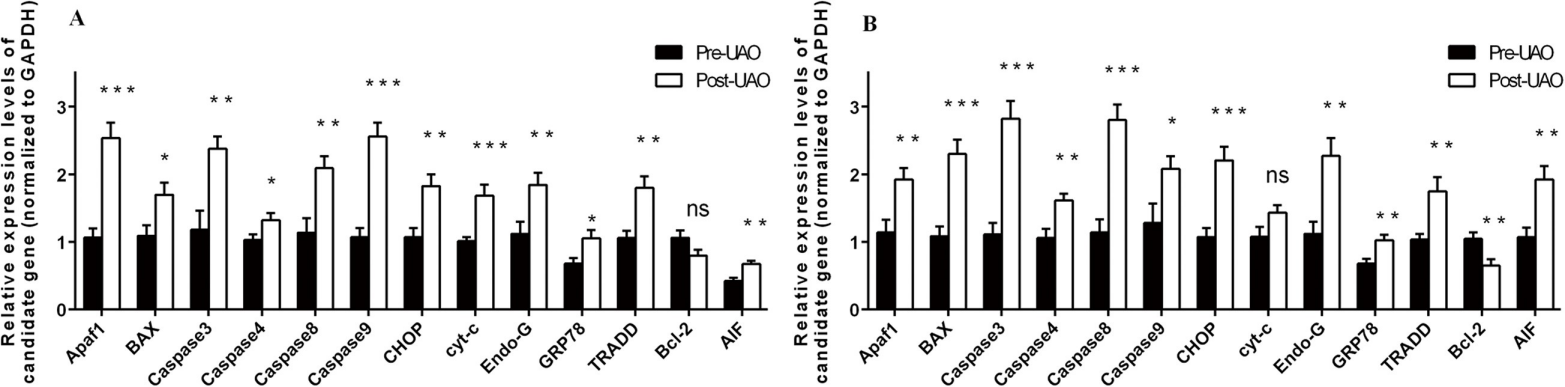
The mRNA expression of apoptosis-related factors in EuE and EE before and after LUAO. (A) After LUAO, the mRNA expression of Apaf1, BAX, caspase-3, caspase-4, caspase-8, caspase-9, CHOP, cyt-c, Endo-G, TRADD, AIF and GRP78 levels of EuE tissue was increased, and mRNA expression of Bcl-2 was down-regulated. (B) After LUAO, the Mrna expression of Apaf1, BAX, caspase-3, caspase-4, caspase-8, caspase-9, CHOP, cyt-c, Endo-G, TRADD, AIF and GRP78 was up-regulated in EE tissues, and the mRNA expression of Bcl-2 levels decreased (^*^P<0.05; ^**^P<0.01; ^***^P < 0.005; ns indicates that P> 0.05).

RT-PCR was used to detect the gene expression of Apaf-1, CHOP, TRADD, Bcl-2, Bax, Endo-G, Cyt-c, AIF, GRP78, Caspase3, Caspase4, Caspase8 and Caspase-9 in 10. As shown in Fig 2, the expression level of apoptosis-related factor Bax, AIF, caspase-3, caspase-4, caspase-8, caspase-9, Endo-G, GRP78, Apf-1, CHOP and TRADD was increased significantly in EuE and EE after LUAO (p<0.05. After LUAO, cyt-c gene expression was increased significantly in EuE (P < 0.005), but no significant increase in EE (P>0.05); the expression of apoptosis-inhibiting gene Bcl2 was decreased significantly in the EE after LUAO (P < 0.01), but no significant decreased in EuE (P>0.05), suggesting that LUAO could induce an apoptosis effect in EuE and EE.

### Western blot results

**Fig 3:**
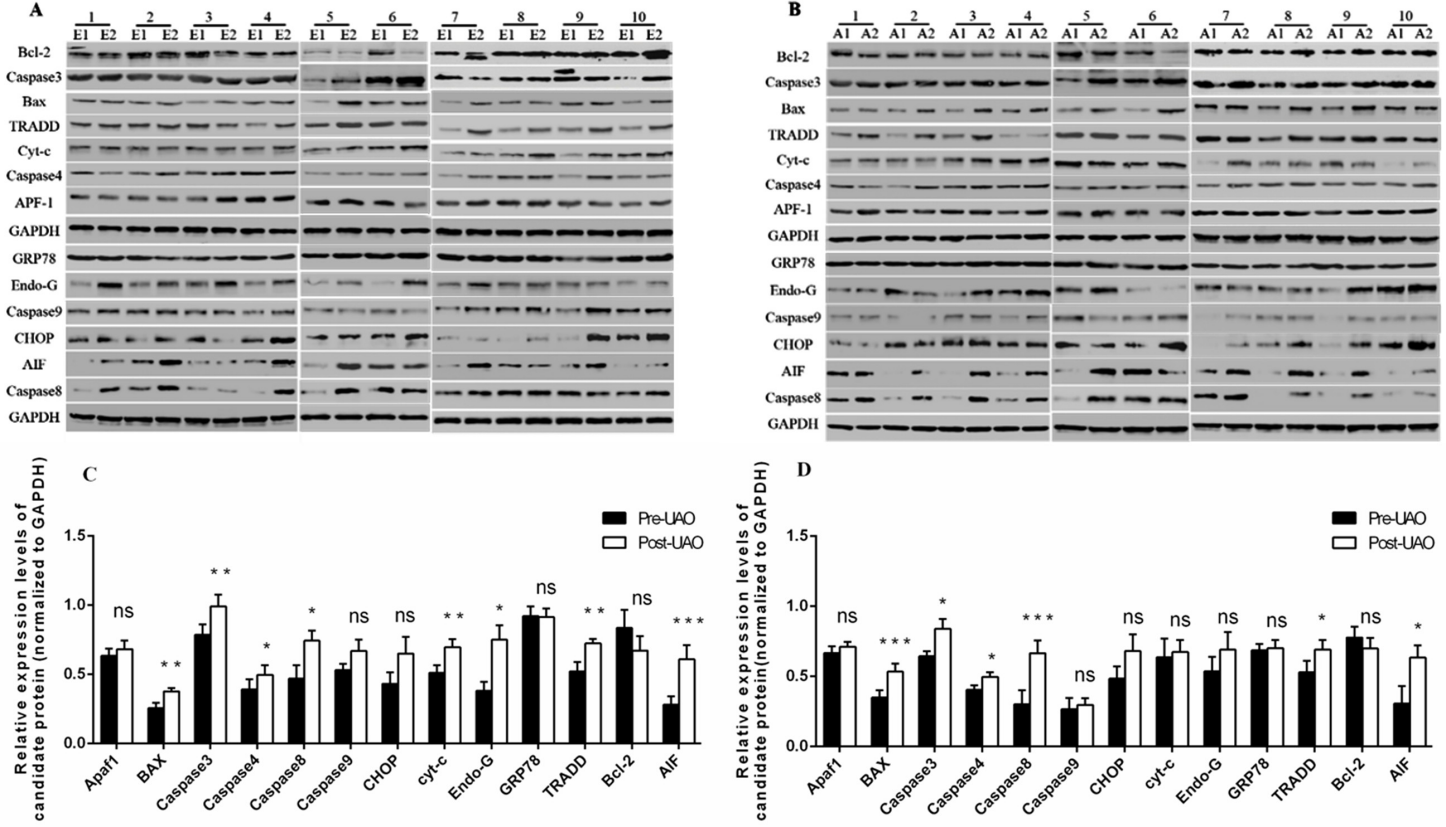
Western Blot showing the protein expression of apoptosis-related factors in EuE and EE before and after LUAO. (A) Difference in protein expression of apoptosis-related factors in EuE tissues before and after LUAO. (B) Difference in proteins expression of apoptosis-related factors in EE tissues before and after LUAO. (C) Corresponding grey value results in EuE Bax, caspase-3, caspase-4, caspase-8, cyt-c, Endo-G, TRADD and AIF protein expression levels were significantly increased after LUAO. (D) Corresponding grey value results in EE Bax, caspase-3, caspase-4, caspase-8, TRADD and AIF protein expression levels were significantly increased after LUAO. (E1/E2: Adenomyotic Eue abtained before and after LUAO; A1/A2: Adenomyotic EE abtained before and after LUAO. The numbers on the head of E1/E2(A1/A2) represent the 10 samples selected in this study. ^*^P<0.05; ^**^P<0.01; ^***^P < 0.005; ns indicates P> 0.05).

Western blot was performed to detect the protein expression of apoptosis-related factors in EuE and EE tissues after LUAO in the 10 specimens. EuE and EE tissues obtained before LUAO were used as the control group, and those obtained after LUAO were used as the experimental group. The protein expression of Bax, AIF, caspase-3, caspase-4, caspase-8, caspase-9, GRP78, Apf-1, CHOP and TRADD was detected by Western blot. (Fig 3).

### Validation of the protein expression level of the apoptosis-related factors in vitro

Primary NESCs, EuESCs and EESCs were cultured for in vitro experiments. The detailed procedures for the isolation, identification and cultivation of the NESCs, EuESCs and EESCs were described previously [18].

**Fig 4:**
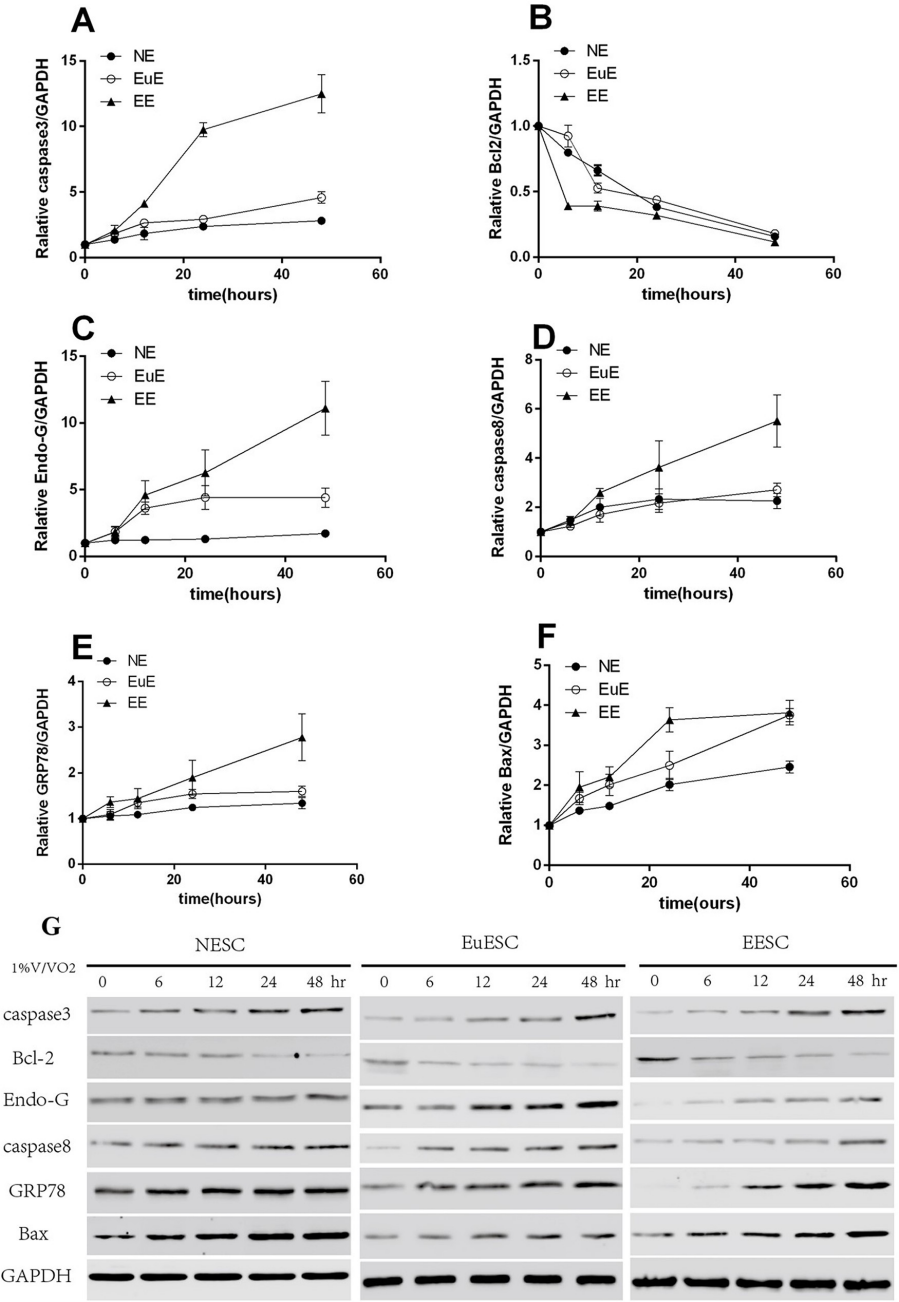
Western Blot showing correlations between protein expression of the apoptosis-related factors. The protein expression of NESC, EuESC, EESC caspase-3 (A), Endo-G (C), caspase-8 (D), GRP78 (E) and Bax (F) was positive correlated with the duration of hypoxia in a time-dependent manner, while the protein expression of Bcl2 (B) was negatively correlated with the duration of hypoxia. (G) Western blot showing the protein expression of the apoptosis-related factors.

Western blot was used to detect the apoptosis-related factors. The result showed that the protein expression levels of apoptosis-related molecules (caspase-3, Bcl2, Bax, EndoG, caspase-8 and GRP78) in NESCs, EuESCs, EESCs was increased significantly at 0, 6, 12, 24 and 48h of hypoxia in a time-dependent manner (Fig 4).

## Discussion

In the present study, we used LUAO to treat patients with adenomyosis and observed changes of EuE and EE tissues by electron microscopy. The result showed that EuE and EE tissues underwent apoptosis after LUAO, presenting typical apoptosis characteristics such as swollen mitochondria, cavitation sample change, nuclear chromatin edge, cell membrane reflex, cytoplasmic concentration and the formation of apoptotic bodies. Then we found the expression of apoptosis-related factors in EuE and EE were increased significantly after LUAO and under hypoxic conditions in vitro.

The main purpose of the current clinical treatment of uterine adenomyosis is to remove the lesions, relieve the symptoms, reduce recurrence, spare the uterus and maintain fertility. By using LUAO combined partial resection, we have achieved good outcome in the clinical treatment of adenomyosis in recent years [12,13]. Our practice and experience suggest that use of the ‘Single organ uterus shock’ model is safe and effective in the treatment of adenomyosis. Nevertheless, the action mechanism of this novel therapy has not yet been validated. We hypothesized that LUAO might involve a complex molecular mechanism. In our previous study [14,19], we preliminarily proposed a ‘Single organ uterus shock’ model with LUAO treatment of uterine fibroids, and supposed that the ischemia hypoxia uterus would experience a transient shock after LUAO, resulting in pathological changes, during which mitochondrial apoptotic pathways were initiated, and apoptosis occurred in myometrial cells due to ischemia hypoxia, which compensated for the uterine blood supply. As a result reperfusion began in the ischemic muscular tissue, which helped restore the physiologic activity gradually. As fibroids and smooth muscle tissue have different hypoxia tolerance, irreversible cell apoptosis and death only occurred in the fibroid tissue, and cells in the uterus still survived [14,19]. Endogenous (mitochondrial) and exogenous approaches represent a classic cell apoptosis pathway, and the main cell apoptosis molecular signalling pathways are described elsewhere [15].

In the ischemic anoxia-induced intrinsic apoptotic pathway, mitochondria transmit different signals to induce stress, such as molecules from the Bcl-2 and Bax families, to maintain the stability of the membrane. When this balance is impaired, apoptosis-related proteins, such as cyt-c, AIF, and Endo-G, are released from the mitochondria, and cyt-c is transferred into the mitochondrial electron transport chain and released into the cytoplasm where it can bind to Apaf-1 to initiate a downstream caspase cascade, activating apoptosis [20]. In addition, Endo-G, an AIF protein, can directly translocate into the nucleus, causing chromosomal shrinkage of DNA fragments, triggering apoptosis [21]. Thus, we selected Bcl-2, Bax, cyt-c, AIF, caspase-3, caspase-9, Endo-G, and Apaf-1 for analysis. In recent years, evidence has shown that endoplasmic reticulum stress was also involved in the apoptosis pathway, as GRP78 promotes apoptosis, and activated caspase-12 can be translocated into the cytoplasm, resulting in the initiation of the caspase-3 cascade response; CHOP can also directly signal through the nucleus in response to apoptosis [22]. Next, we detected GRP78 and CHOP expression and determined the expression of exogenous channel apoptosis-related proteins caspase-4, TRADD, and caspase-8. Analysis of protein expression revealed that the expression of Bcl-2, Bax, cyt-c, AIF, caspase-3, caspase-9, Endo-G and Apaf-1 was significantly increased, prompting mitochondrial apoptosis, which plays a major role in adenomyosis after UAO treatment, and inducing irreversible apoptosis in many cells, which is consistent with the mechanism shown in elsewhere [15]. It can be concluded that UAO mainly initiates the mitochondrial pathway, accompanied by the expression of endoplasmic reticulum stress factors, causing cellular apoptosis. Our previous research on LUAO treatment of hysteromyoma showed that there is more sensitivity in uterine fibroids in relatively smooth muscle tissue under hypoxic conditions, and more apoptosis, mainly involving the activation of the endogenous mitochondrial apoptosis pathway, occurs during endoplasmic reticulum stress, thereby releasing signalling molecules to activate the caspase signalling cascade, inducing apoptosis [19,23]. The results are consistent with our experimental conclusions.

We constructed a cell hypoxia model in vitro to determine the anoxic conditions at 0, 6, 12, 24 and 48h and selected apoptosis-related proteins in the mitochondria and endoplasmic reticulum to confirm the results. In CESC, EuESC and EESC cells, changes in the expression of caspase-3, Endo-G, caspase-8, GRP78 and Bax showed a positive correlation with the duration of anoxia, particularly for caspase-3, Endo-G and caspase-8 in adenomyosis. However, the expression of Bcl-2 was negatively correlated with hypoxia and inhibited expression. Cytological experiments with prolonged hypoxia confirmed the apoptotic changes and showed a significant difference. The cytology experiments also revealed that with prolonged hypoxia, more significant changes in apoptosis were observed, and these changes were more obvious in the adenomyotic EuE and EE than those in the normal endometrium, although the mechanism remains to be further elucidated. This phenomenon may be correlated with our LUAO solution because the main body combined surgical treatment had a good clinical effect in uterine adenomyosis. However, the effect of LUAO joint lesion resection treatment of adenomyosis on ovarian and reproductive function has been debated. In the early stage, we selected LUAO preoperative patients and followed them up postoperatively for 1, 3 and 6 months to assess the impact of FSH, LH, E2, and INHB (serum inhibin B) on ovarian function, and the results showed no significant difference [24].

In summary, Our study confirms that the EE has experienced more significant apoptosis after LUAO, which may clarify the precise mechanisms so as to lay a theoretical basis for the clinical treatment of the adenomyosis. However, there is still a need for further study of molecular mechanism. In addition, the comprehensive curative effect of this new method and its impact on ovarian function and fertility need to be confirmed in large multicenter randomized clinical trials.

## Supporting information

S1 Table The relative m RNA expression of apoptosis-related facors in EuE and EE before and after LUAO in 10 cases. E1/E2: EuE abtained before and after LUAO; A1/A2: EE abtained before and after LUAO.
(DOCX)

S2 Table Western Blot grey value of the protein expression of apoptosis-relatated factors in EuE and EE before and after LUAO in 10 cases. (DOCX)

S3 Table Western Blot grey value of the protein expression of apoptosis-related factors in primary NESCs, EuESCs and EESCs at 0, 6, 12, 24, 48h of hypoxia in 3 cases. (DOCX)
