# Supplementary material for: Eutopic/ectopic endometrial apoptosis initiated by bilateral uterine artery occlusion: A new therapeutic mechanism for uterus-sparing surgery in adenomyosis
Source: PLoS One. 2017 Apr 13;12(4):e0175511. doi: 10.1371/journal.pone.0175511 (PMC5391022; doi:10.1371/journal.pone.0175511)
Supplement: S1 Table — E1/E2: EuE abtained before and after LUAO; A1/A2: EE abtained before and after LUAO. (DOCX) [file pone.0175511.s001.docx]

|  |  |  |  |  |  |  |  |  |  |  |
| --- | --- | --- | --- | --- | --- | --- | --- | --- | --- | --- |
|  |  |  | Ct | mRNA |  |  |  |  | Ct | mRNA |
| BCL-2 | 1 | E1 | 23.26 | 0.03125 |  | BAX | 1 | E1 | 23.72 | 0.022718 |
|  |  | E2 | 24.71 | 0.012344 |  |  |  | E2 | 22.98 | 0.04095 |
|  |  | A1 | 23.72 | 0.029977 |  |  |  | A1 | 25.03 | 0.01209 |
|  |  | A2 | 24.7 | 0.019505 |  |  |  | A2 | 23.04 | 0.06164 |
|  | 2 | E1 | 23.17 | 0.025033 |  |  | 2 | E1 | 24.12 | 0.012958 |
|  |  | E2 | 24.34 | 0.01278 |  |  |  | E2 | 23.08 | 0.030607 |
|  |  | A1 | 23.96 | 0.033262 |  |  |  | A1 | 24.32 | 0.025916 |
|  |  | A2 | 24.14 | 0.011049 |  |  |  | A2 | 22.19 | 0.042689 |
|  | 3 | E1 | 23.33 | 0.028557 |  |  | 3 | E1 | 23.12 | 0.033032 |
|  |  | E2 | 24.72 | 0.01468 |  |  |  | E2 | 22.85 | 0.05366 |
|  |  | A1 | 23.07 | 0.023035 |  |  |  | A1 | 22.08 | 0.045753 |
|  |  | A2 | 24.28 | 0.011049 |  |  |  | A2 | 21.65 | 0.068393 |
|  | 4 | E1 | 22.68 | 0.030607 |  |  | 4 | E1 | 23 | 0.024518 |
|  |  | E2 | 23.33 | 0.01937 |  |  |  | E2 | 22.02 | 0.048027 |
|  |  | A1 | 23.35 | 0.028164 |  |  |  | A1 | 22.99 | 0.036147 |
|  |  | A2 | 23.78 | 0.024349 |  |  |  | A2 | 22.23 | 0.071298 |
|  | 5 | E1 | 24.08 | 0.009958 |  |  | 5 | E1 | 22.76 | 0.024861 |
|  |  | E2 | 24.16 | 0.01209 |  |  |  | E2 | 22.96 | 0.027776 |
|  |  | A1 | 22.83 | 0.035403 |  |  |  | A1 | 23.45 | 0.023035 |
|  |  | A2 | 24.28 | 0.016064 |  |  |  | A2 | 22.79 | 0.045123 |
|  | 6 | E1 | 23.22 | 0.027394 |  |  | 6 | E1 | 22.07 | 0.060791 |
|  |  | E2 | 24.06 | 0.015843 |  |  |  | E2 | 22.16 | 0.059129 |
|  |  | A1 | 24.18 | 0.012344 |  |  |  | A1 | 22.78 | 0.032577 |
|  |  | A2 | 23.04 | 0.026278 |  |  |  | A2 | 21.64 | 0.069348 |
|  | 7 | E1 | 24.43 | 0.014279 |  |  | 7 | E1 | 22.99 | 0.038741 |
|  |  | E2 | 23.13 | 0.025208 |  |  |  | E2 | 22.15 | 0.049721 |
|  |  | A1 | 23.25 | 0.031034 |  |  |  | A1 | 24.28 | 0.015198 |
|  |  | A2 | 23.77 | 0.018972 |  |  |  | A2 | 22.03 | 0.063372 |
|  | 8 | E1 | 23.22 | 0.018199 |  |  | 8 | E1 | 22.42 | 0.031686 |
|  |  | E2 | 24.67 | 0.013508 |  |  |  | E2 | 23.65 | 0.027394 |
|  |  | A1 | 23.6 | 0.033726 |  |  |  | A1 | 24.58 | 0.017098 |
|  |  | A2 | 23.24 | 0.026461 |  |  |  | A2 | 23.51 | 0.021944 |
|  | 9 | E1 | 23.56 | 0.017948 |  |  | 9 | E1 | 22.63 | 0.034197 |
|  |  | E2 | 23.45 | 0.027584 |  |  |  | E2 | 21.99 | 0.075887 |
|  |  | A1 | 23.18 | 0.017824 |  |  |  | A1 | 23.15 | 0.018199 |
|  |  | A2 | 24.64 | 0.006087 |  |  |  | A2 | 21.85 | 0.042101 |
|  | 10 | E1 | 23.8 | 0.019237 |  |  | 10 | E1 | 24.04 | 0.016289 |
|  |  | E2 | 24.23 | 0.01418 |  |  |  | E2 | 22.32 | 0.05329 |
|  |  | A1 | 23.1 | 0.023035 |  |  |  | A1 | 22.6 | 0.032577 |
|  |  | A2 | 25.5 | 0.006172 |  |  |  | A2 | 22.15 | 0.062935 |
|  |  |  |  |  |  |  |  |  |  |  |
|  |  |  |  |  |  |  |  |  |  |  |
|  |  |  | Ct | mRNA |  |  |  |  | Ct | mRNA |
| caspase3 | 1 | E1 | 25.57 | 0.006302 |  | caspase4 | 1 | E1 | 23.97 | 0.019104 |
|  |  | E2 | 24.62 | 0.013139 |  |  |  | E2 | 23.24 | 0.034197 |
|  |  | A1 | 26.37 | 0.004776 |  |  |  | A1 | 24.59 | 0.016402 |
|  |  | A2 | 24.98 | 0.016064 |  |  |  | A2 | 24.72 | 0.019237 |
|  | 2 | E1 | 25.65 | 0.004487 |  |  | 2 | E1 | 23.48 | 0.020193 |
|  |  | E2 | 24.63 | 0.010453 |  |  |  | E2 | 23.02 | 0.031907 |
|  |  | A1 | 25.93 | 0.00849 |  |  |  | A1 | 25.02 | 0.015953 |
|  |  | A2 | 23.42 | 0.018199 |  |  |  | A2 | 22.17 | 0.043285 |
|  | 3 | E1 | 25.08 | 0.00849 |  |  | 3 | E1 | 23.43 | 0.026645 |
|  |  | E2 | 24.22 | 0.020761 |  |  |  | E2 | 24.52 | 0.016863 |
|  |  | A1 | 23.94 | 0.012604 |  |  |  | A1 | 22.16 | 0.043285 |
|  |  | A2 | 22.99 | 0.027017 |  |  |  | A2 | 22.67 | 0.033726 |
|  | 4 | E1 | 24.88 | 0.006661 |  |  | 4 | E1 | 22.99 | 0.024689 |
|  |  | E2 | 23 | 0.024349 |  |  |  | E2 | 22.38 | 0.037421 |
|  |  | A1 | 24.75 | 0.010672 |  |  |  | A1 | 23.55 | 0.024518 |
|  |  | A2 | 23.44 | 0.03082 |  |  |  | A2 | 23.31 | 0.033726 |
|  | 5 | E1 | 24.57 | 0.00709 |  |  | 5 | E1 | 22.83 | 0.023683 |
|  |  | E2 | 23.51 | 0.018972 |  |  |  | E2 | 22.91 | 0.028756 |
|  |  | A1 | 25.35 | 0.006172 |  |  |  | A1 | 23.78 | 0.018326 |
|  |  | A2 | 23.98 | 0.019777 |  |  |  | A2 | 23.48 | 0.02797 |
|  | 6 | E1 | 23.08 | 0.030186 |  |  | 6 | E1 | 23.62 | 0.020761 |
|  |  | E2 | 23.42 | 0.024689 |  |  |  | E2 | 23.12 | 0.030395 |
|  |  | A1 | 23.9 | 0.014989 |  |  |  | A1 | 23.76 | 0.016516 |
|  |  | A2 | 22.91 | 0.028756 |  |  |  | A2 | 22.44 | 0.03983 |
|  | 7 | E1 | 24.83 | 0.010821 |  |  | 7 | E1 | 24.13 | 0.017579 |
|  |  | E2 | 23.3 | 0.022406 |  |  |  | E2 | 22.75 | 0.032804 |
|  |  | A1 | 25.7 | 0.00568 |  |  |  | A1 | 23.86 | 0.020333 |
|  |  | A2 | 23.03 | 0.031686 |  |  |  | A2 | 23.05 | 0.03125 |
|  | 8 | E1 | 24.54 | 0.007289 |  |  | 8 | E1 | 23.52 | 0.014782 |
|  |  | E2 | 23.93 | 0.022561 |  |  |  | E2 | 24.01 | 0.021344 |
|  |  | A1 | 26.07 | 0.006087 |  |  |  | A1 | 24.66 | 0.016176 |
|  |  | A2 | 24.36 | 0.012174 |  |  |  | A2 | 22.97 | 0.031907 |
|  | 9 | E1 | 23.83 | 0.014885 |  |  | 9 | E1 | 22.63 | 0.034197 |
|  |  | E2 | 23.69 | 0.023357 |  |  |  | E2 | 24.05 | 0.018199 |
|  |  | A1 | 25.09 | 0.004743 |  |  |  | A1 | 22.39 | 0.03082 |
|  |  | A2 | 22.94 | 0.019777 |  |  |  | A2 | 21.95 | 0.039282 |
|  | 10 | E1 | 25.61 | 0.005486 |  |  | 10 | E1 | 23.94 | 0.017458 |
|  |  | E2 | 23.5 | 0.023519 |  |  |  | E2 | 23.16 | 0.02977 |
|  |  | A1 | 23.55 | 0.016863 |  |  |  | A1 | 23.48 | 0.017701 |
|  |  | A2 | 23.35 | 0.027394 |  |  |  | A2 | 23.01 | 0.034674 |
|  |  |  |  |  |  |  |  |  |  |  |
|  |  |  |  |  |  |  |  |  |  |  |
|  |  |  | Ct | mRNA |  |  |  |  | Ct | mRNA |
| caspase8 | 1 | E1 | 25.07 | 0.008912 |  | caspase9 | 1 | E1 | 27 | 0.002339 |
|  |  | E2 | 23.93 | 0.021197 |  |  |  | E2 | 25.8 | 0.005799 |
|  |  | A1 | 24.89 | 0.013322 |  |  |  | A1 | 27.28 | 0.002542 |
|  |  | A2 | 24.74 | 0.018972 |  |  |  | A2 | 25.99 | 0.007977 |
|  | 2 | E1 | 26.01 | 0.003496 |  |  | 2 | E1 | 25.21 | 0.006087 |
|  |  | E2 | 24.81 | 0.009227 |  |  |  | E2 | 24.53 | 0.011203 |
|  |  | A1 | 26.29 | 0.006615 |  |  |  | A1 | 25.96 | 0.008315 |
|  |  | A2 | 23.41 | 0.018326 |  |  |  | A2 | 23.92 | 0.012869 |
|  | 3 | E1 | 25.22 | 0.007705 |  |  | 3 | E1 | 26.38 | 0.003448 |
|  |  | E2 | 24.4 | 0.018326 |  |  |  | E2 | 25.11 | 0.011203 |
|  |  | A1 | 24.19 | 0.010598 |  |  |  | A1 | 24.87 | 0.006615 |
|  |  | A2 | 23.33 | 0.021344 |  |  |  | A2 | 24.99 | 0.006754 |
|  | 4 | E1 | 25.07 | 0.005839 |  |  | 4 | E1 | 26.33 | 0.002438 |
|  |  | E2 | 24.27 | 0.010097 |  |  |  | E2 | 25.04 | 0.005921 |
|  |  | A1 | 26.05 | 0.004334 |  |  |  | A1 | 27.14 | 0.002036 |
|  |  | A2 | 24.52 | 0.014579 |  |  |  | A2 | 25.3 | 0.00849 |
|  | 5 | E1 | 24.62 | 0.006848 |  |  | 5 | E1 | 25.49 | 0.003747 |
|  |  | E2 | 23.81 | 0.01541 |  |  |  | E2 | 24.38 | 0.01038 |
|  |  | A1 | 25.42 | 0.00588 |  |  |  | A1 | 24.34 | 0.01243 |
|  |  | A2 | 24.26 | 0.016289 |  |  |  | A2 | 25.02 | 0.009618 |
|  | 6 | E1 | 23.41 | 0.024014 |  |  | 6 | E1 | 26.78 | 0.002323 |
|  |  | E2 | 23.74 | 0.019777 |  |  |  | E2 | 24.76 | 0.009753 |
|  |  | A1 | 24.04 | 0.013602 |  |  |  | A1 | 24.09 | 0.013139 |
|  |  | A2 | 22.98 | 0.027394 |  |  |  | A2 | 23.99 | 0.013602 |
|  | 7 | E1 | 25.03 | 0.00942 |  |  | 7 | E1 | 25.91 | 0.005119 |
|  |  | E2 | 23.23 | 0.023519 |  |  |  | E2 | 24.89 | 0.007442 |
|  |  | A1 | 26.24 | 0.003906 |  |  |  | A1 | 25.26 | 0.007705 |
|  |  | A2 | 23.59 | 0.021493 |  |  |  | A2 | 24.31 | 0.013048 |
|  | 8 | E1 | 24.07 | 0.010097 |  |  | 8 | E1 | 24.72 | 0.006434 |
|  |  | E2 | 24.11 | 0.019915 |  |  |  | E2 | 24.74 | 0.012869 |
|  |  | A1 | 26.48 | 0.004581 |  |  |  | A1 | 27.38 | 0.002455 |
|  |  | A2 | 24.52 | 0.010896 |  |  |  | A2 | 25.48 | 0.005601 |
|  | 9 | E1 | 23.98 | 0.013415 |  |  | 9 | E1 | 26.25 | 0.002781 |
|  |  | E2 | 23.89 | 0.020333 |  |  |  | E2 | 24.91 | 0.010027 |
|  |  | A1 | 25.33 | 0.004016 |  |  |  | A1 | 25.84 | 0.00282 |
|  |  | A2 | 22.5 | 0.02683 |  |  |  | A2 | 24.05 | 0.009163 |
|  | 10 | E1 | 25.36 | 0.006524 |  |  | 10 | E1 | 25.93 | 0.004395 |
|  |  | E2 | 23.78 | 0.01937 |  |  |  | E2 | 24.91 | 0.008851 |
|  |  | A1 | 23.82 | 0.013985 |  |  |  | A1 | 26.53 | 0.002137 |
|  |  | A2 | 23.62 | 0.022718 |  |  |  | A2 | 24.69 | 0.010821 |
|  |  |  |  |  |  |  |  |  |  |  |
|  |  |  |  |  |  |  |  |  |  |  |
|  |  |  | Ct | mRNA |  |  |  |  | Ct | mRNA |
| cyt-c | 1 | E1 | 26.11 | 0.004334 |  | TRADD | 1 | E1 | 22.8 | 0.042986 |
|  |  | E2 | 25.13 | 0.009227 |  |  |  | E2 | 22.05 | 0.078021 |
|  |  | A1 | 25.93 | 0.006479 |  |  |  | A1 | 22.9 | 0.052922 |
|  |  | A2 | 25.95 | 0.008201 |  |  |  | A2 | 22.25 | 0.106579 |
|  | 2 | E1 | 25.3 | 0.005719 |  |  | 2 | E1 | 23.02 | 0.027776 |
|  |  | E2 | 24.58 | 0.010821 |  |  |  | E2 | 22.51 | 0.045437 |
|  |  | A1 | 26.62 | 0.005263 |  |  |  | A1 | 23.97 | 0.033032 |
|  |  | A2 | 24.29 | 0.009958 |  |  |  | A2 | 21.08 | 0.092142 |
|  | 3 | E1 | 26.1 | 0.004187 |  |  | 3 | E1 | 23.25 | 0.030186 |
|  |  | E2 | 26.32 | 0.004843 |  |  |  | E2 | 22.76 | 0.057114 |
|  |  | A1 | 25.34 | 0.004776 |  |  |  | A1 | 22.43 | 0.035897 |
|  |  | A2 | 24.66 | 0.00849 |  |  |  | A2 | 21.36 | 0.08362 |
|  | 4 | E1 | 25.52 | 0.004275 |  |  | 4 | E1 | 22.7 | 0.030186 |
|  |  | E2 | 24.35 | 0.009552 |  |  |  | E2 | 21.27 | 0.080772 |
|  |  | A1 | 25.68 | 0.005601 |  |  |  | A1 | 22.2 | 0.0625 |
|  |  | A2 | 25.05 | 0.010097 |  |  |  | A2 | 23.66 | 0.026461 |
|  | 5 | E1 | 25.01 | 0.005226 |  |  | 5 | E1 | 23.4 | 0.015953 |
|  |  | E2 | 24.17 | 0.012007 |  |  |  | E2 | 22.14 | 0.049037 |
|  |  | A1 | 25.61 | 0.005154 |  |  |  | A1 | 22.33 | 0.050067 |
|  |  | A2 | 24.93 | 0.010237 |  |  |  | A2 | 21.86 | 0.085971 |
|  | 6 | E1 | 25.83 | 0.004487 |  |  | 6 | E1 | 22.96 | 0.032804 |
|  |  | E2 | 25.85 | 0.004581 |  |  |  | E2 | 21.98 | 0.066986 |
|  |  | A1 | 24.35 | 0.010972 |  |  |  | A1 | 23.11 | 0.025916 |
|  |  | A2 | 24.61 | 0.008851 |  |  |  | A2 | 21.29 | 0.088388 |
|  | 7 | E1 | 25.49 | 0.006848 |  |  | 7 | E1 | 22.51 | 0.054034 |
|  |  | E2 | 24.46 | 0.010027 |  |  |  | E2 | 23.04 | 0.02683 |
|  |  | A1 | 24.64 | 0.011842 |  |  |  | A1 | 23.12 | 0.03396 |
|  |  | A2 | 24.54 | 0.011125 |  |  |  | A2 | 21.87 | 0.070805 |
|  | 8 | E1 | 24.95 | 0.005486 |  |  | 8 | E1 | 21.65 | 0.054034 |
|  |  | E2 | 25.87 | 0.00588 |  |  |  | E2 | 22 | 0.085971 |
|  |  | A1 | 27.19 | 0.002801 |  |  |  | A1 | 23.1 | 0.047696 |
|  |  | A2 | 25.38 | 0.006003 |  |  |  | A2 | 23.36 | 0.024349 |
|  | 9 | E1 | 25.12 | 0.006087 |  |  | 9 | E1 | 22.61 | 0.034674 |
|  |  | E2 | 24.92 | 0.009958 |  |  |  | E2 | 21.98 | 0.076415 |
|  |  | A1 | 24.68 | 0.006302 |  |  |  | A1 | 21.71 | 0.049378 |
|  |  | A2 | 25.16 | 0.004245 |  |  |  | A2 | 21.02 | 0.074842 |
|  | 10 | E1 | 25.8 | 0.004809 |  |  | 10 | E1 | 22.62 | 0.043586 |
|  |  | E2 | 24.94 | 0.008669 |  |  |  | E2 | 22.2 | 0.057912 |
|  |  | A1 | 24.99 | 0.006215 |  |  |  | A1 | 21.96 | 0.050766 |
|  |  | A2 | 24.85 | 0.009685 |  |  |  | A2 | 21.55 | 0.095391 |
|  |  |  |  |  |  |  |  |  |  |  |
|  |  |  |  |  |  |  |  |  |  |  |
|  |  |  | Ct | mRNA |  |  |  |  | Ct | mRNA |
| GPR78 | 1 | E1 | 23.5 | 0.028557 |  | CHOP | 1 | E1 | 24.06 | 0.017948 |
|  |  | E2 | 22.75 | 0.044502 |  |  |  | E2 | 23.41 | 0.030395 |
|  |  | A1 | 24.55 | 0.021642 |  |  |  | A1 | 24.35 | 0.01937 |
|  |  | A2 | 22.66 | 0.0625 |  |  |  | A2 | 23.78 | 0.036906 |
|  | 2 | E1 | 23.53 | 0.022406 |  |  | 2 | E1 | 24.32 | 0.011281 |
|  |  | E2 | 22.26 | 0.047039 |  |  |  | E2 | 24.46 | 0.01176 |
|  |  | A1 | 22.59 | 0.032352 |  |  |  | A1 | 25.08 | 0.015303 |
|  |  | A2 | 22.99 | 0.065154 |  |  |  | A2 | 23.32 | 0.019505 |
|  | 3 | E1 | 24.18 | 0.021344 |  |  | 3 | E1 | 24.22 | 0.01541 |
|  |  | E2 | 23.18 | 0.031686 |  |  |  | E2 | 23.52 | 0.033726 |
|  |  | A1 | 22.75 | 0.031907 |  |  |  | A1 | 23.23 | 0.020617 |
|  |  | A2 | 22.05 | 0.046714 |  |  |  | A2 | 22.25 | 0.045123 |
|  | 4 | E1 | 22.39 | 0.037163 |  |  | 4 | E1 | 24.18 | 0.010821 |
|  |  | E2 | 22.06 | 0.047039 |  |  |  | E2 | 23.41 | 0.018326 |
|  |  | A1 | 22.4 | 0.063372 |  |  |  | A1 | 24.6 | 0.011842 |
|  |  | A2 | 22.74 | 0.042986 |  |  |  | A2 | 23.74 | 0.025033 |
|  | 5 | E1 | 22.91 | 0.028756 |  |  | 5 | E1 | 23.77 | 0.012344 |
|  |  | E2 | 21.99 | 0.042394 |  |  |  | E2 | 22.63 | 0.034915 |
|  |  | A1 | 22.95 | 0.040386 |  |  |  | A1 | 24.49 | 0.011203 |
|  |  | A2 | 21.99 | 0.063372 |  |  |  | A2 | 23.26 | 0.032577 |
|  | 6 | E1 | 22.36 | 0.051474 |  |  | 6 | E1 | 22.94 | 0.033262 |
|  |  | E2 | 21.52 | 0.089003 |  |  |  | E2 | 22.95 | 0.034197 |
|  |  | A1 | 21.85 | 0.059954 |  |  |  | A1 | 22.98 | 0.02836 |
|  |  | A2 | 22.05 | 0.054034 |  |  |  | A2 | 22.71 | 0.033032 |
|  | 7 | E1 | 22.37 | 0.042689 |  |  | 7 | E1 | 24.02 | 0.018972 |
|  |  | E2 | 21.98 | 0.078021 |  |  |  | E2 | 22.62 | 0.035897 |
|  |  | A1 | 22.36 | 0.050415 |  |  |  | A1 | 24.66 | 0.011679 |
|  |  | A2 | 21.79 | 0.085378 |  |  |  | A2 | 22.45 | 0.047366 |
|  | 8 | E1 | 22.86 | 0.047366 |  |  | 8 | E1 | 23.06 | 0.020333 |
|  |  | E2 | 22.06 | 0.040667 |  |  |  | E2 | 23.09 | 0.040386 |
|  |  | A1 | 22.83 | 0.035158 |  |  |  | A1 | 25.18 | 0.011281 |
|  |  | A2 | 22.74 | 0.061214 |  |  |  | A2 | 23.75 | 0.018581 |
|  | 9 | E1 | 23.58 | 0.025208 |  |  | 9 | E1 | 22.84 | 0.029564 |
|  |  | E2 | 22.24 | 0.044811 |  |  |  | E2 | 22.8 | 0.043285 |
|  |  | A1 | 22.08 | 0.035897 |  |  |  | A1 | 24.24 | 0.008549 |
|  |  | A2 | 20.98 | 0.0819 |  |  |  | A2 | 21.99 | 0.038208 |
|  | 10 | E1 | 23.94 | 0.017337 |  |  | 10 | E1 | 24.08 | 0.015843 |
|  |  | E2 | 22.97 | 0.034197 |  |  |  | E2 | 22.96 | 0.034197 |
|  |  | A1 | 23.2 | 0.030395 |  |  |  | A1 | 23.09 | 0.023196 |
|  |  | A2 | 22.14 | 0.044811 |  |  |  | A2 | 22.98 | 0.035403 |
|  |  |  |  |  |  |  |  |  |  |  |
|  |  |  |  |  |  |  |  |  |  |  |
|  |  |  | Ct | mRNA |  |  |  |  | Ct | mRNA |
| Apaf1 | 1 | E1 | 26.59 | 0.003108 |  | AIF | 1 | E1 | 26.46 | 0.003401 |
|  |  | E2 | 25.74 | 0.006045 |  |  |  | E2 | 25.62 | 0.00657 |
|  |  | A1 | 26.7 | 0.003799 |  |  |  | A1 | 26.84 | 0.003448 |
|  |  | A2 | 26.12 | 0.007289 |  |  |  | A2 | 25.63 | 0.010237 |
|  | 2 | E1 | 25.56 | 0.004776 |  |  | 2 | E1 | 25.14 | 0.00639 |
|  |  | E2 | 24.29 | 0.01323 |  |  |  | E2 | 25.4 | 0.00613 |
|  |  | A1 | 26.08 | 0.007652 |  |  |  | A1 | 26.13 | 0.007391 |
|  |  | A2 | 23.92 | 0.012869 |  |  |  | A2 | 24.65 | 0.007759 |
|  | 3 | E1 | 26.46 | 0.003262 |  |  | 3 | E1 | 25.84 | 0.005013 |
|  |  | E2 | 25.48 | 0.008669 |  |  |  | E2 | 25.29 | 0.009889 |
|  |  | A1 | 24.69 | 0.007494 |  |  |  | A1 | 24.52 | 0.008431 |
|  |  | A2 | 25.03 | 0.00657 |  |  |  | A2 | 24.24 | 0.011359 |
|  | 4 | E1 | 26.4 | 0.002323 |  |  | 4 | E1 | 25.62 | 0.003988 |
|  |  | E2 | 25.43 | 0.004518 |  |  |  | E2 | 24.38 | 0.009355 |
|  |  | A1 | 26.67 | 0.00282 |  |  |  | A1 | 25.57 | 0.006045 |
|  |  | A2 | 25.61 | 0.006848 |  |  |  | A2 | 24.91 | 0.011125 |
|  | 5 | E1 | 25.66 | 0.003331 |  |  | 5 | E1 | 24.32 | 0.008431 |
|  |  | E2 | 24.52 | 0.00942 |  |  |  | E2 | 25.03 | 0.006615 |
|  |  | A1 | 24.73 | 0.009486 |  |  |  | A1 | 26.01 | 0.003906 |
|  |  | A2 | 25.12 | 0.008974 |  |  |  | A2 | 25.73 | 0.00588 |
|  | 6 | E1 | 26.56 | 0.002705 |  |  | 6 | E1 | 25.48 | 0.005719 |
|  |  | E2 | 24.66 | 0.010453 |  |  |  | E2 | 24.73 | 0.009958 |
|  |  | A1 | 24.67 | 0.00879 |  |  |  | A1 | 25.25 | 0.00588 |
|  |  | A2 | 24.36 | 0.010525 |  |  |  | A2 | 24.07 | 0.012869 |
|  | 7 | E1 | 26.01 | 0.004776 |  |  | 7 | E1 | 25.58 | 0.006434 |
|  |  | E2 | 24.8 | 0.007922 |  |  |  | E2 | 24.55 | 0.00942 |
|  |  | A1 | 25.61 | 0.006045 |  |  |  | A1 | 26.56 | 0.003129 |
|  |  | A2 | 24.61 | 0.010598 |  |  |  | A2 | 24.97 | 0.008258 |
|  | 8 | E1 | 24.46 | 0.007705 |  |  | 8 | E1 | 25.02 | 0.005226 |
|  |  | E2 | 24.78 | 0.012517 |  |  |  | E2 | 24.96 | 0.011049 |
|  |  | A1 | 27.1 | 0.002981 |  |  |  | A1 | 26.73 | 0.003852 |
|  |  | A2 | 25.63 | 0.005048 |  |  |  | A2 | 26.28 | 0.003217 |
|  | 9 | E1 | 26.24 | 0.002801 |  |  | 9 | E1 | 25.61 | 0.004334 |
|  |  | E2 | 24.83 | 0.010598 |  |  |  | E2 | 25.52 | 0.00657 |
|  |  | A1 | 25.83 | 0.00284 |  |  |  | A1 | 25.88 | 0.002743 |
|  |  | A2 | 23.67 | 0.011924 |  |  |  | A2 | 24.29 | 0.007759 |
|  | 10 | E1 | 26.06 | 0.004016 |  |  | 10 | E1 | 26.67 | 0.002631 |
|  |  | E2 | 24.88 | 0.009037 |  |  |  | E2 | 25.43 | 0.006172 |
|  |  | A1 | 26.49 | 0.002197 |  |  |  | A1 | 25.78 | 0.003594 |
|  |  | A2 | 24.73 | 0.010525 |  |  |  | A2 | 25.05 | 0.008431 |
|  |  |  |  |  |  |  |  |  |  |  |
|  |  |  |  |  |  |  |  |  |  |  |
|  |  |  | Ct | mRNA |  |  |  |  | Ct |  |
| Endo-G | 1 | E1 | 24.17 | 0.016631 |  | GAPDH | 1 | E1 | 18.26 |  |
|  |  | E2 | 23.35 | 0.031686 |  |  |  | E2 | 18.37 |  |
|  |  | A1 | 25.2 | 0.010746 |  |  |  | A1 | 18.66 |  |
|  |  | A2 | 23.7 | 0.03901 |  |  |  | A2 | 19.02 |  |
|  | 2 | E1 | 23.08 | 0.026645 |  |  | 2 | E1 | 17.85 |  |
|  |  | E2 | 23.33 | 0.025737 |  |  |  | E2 | 18.05 |  |
|  |  | A1 | 24.01 | 0.032129 |  |  |  | A1 | 19.05 |  |
|  |  | A2 | 23.1 | 0.022718 |  |  |  | A2 | 17.64 |  |
|  | 3 | E1 | 23.85 | 0.019915 |  |  | 3 | E1 | 18.2 |  |
|  |  | E2 | 23.3 | 0.039282 |  |  |  | E2 | 18.63 |  |
|  |  | A1 | 22.47 | 0.034915 |  |  |  | A1 | 17.63 |  |
|  |  | A2 | 22.04 | 0.052193 |  |  |  | A2 | 17.78 |  |
|  | 4 | E1 | 23.68 | 0.015303 |  |  | 4 | E1 | 17.65 |  |
|  |  | E2 | 22.36 | 0.037944 |  |  |  | E2 | 17.64 |  |
|  |  | A1 | 23.35 | 0.028164 |  |  |  | A1 | 18.2 |  |
|  |  | A2 | 22.66 | 0.052922 |  |  |  | A2 | 18.42 |  |
|  | 5 | E1 | 24.46 | 0.007652 |  |  | 5 | E1 | 17.43 |  |
|  |  | E2 | 23.74 | 0.016176 |  |  |  | E2 | 17.79 |  |
|  |  | A1 | 24.61 | 0.010309 |  |  |  | A1 | 18.01 |  |
|  |  | A2 | 22.65 | 0.049721 |  |  |  | A2 | 18.32 |  |
|  | 6 | E1 | 22.42 | 0.047696 |  |  | 6 | E1 | 18.03 |  |
|  |  | E2 | 22.59 | 0.043889 |  |  |  | E2 | 18.08 |  |
|  |  | A1 | 23.08 | 0.026461 |  |  |  | A1 | 17.84 |  |
|  |  | A2 | 21.84 | 0.060371 |  |  |  | A2 | 17.79 |  |
|  | 7 | E1 | 23.47 | 0.027776 |  |  | 7 | E1 | 18.3 |  |
|  |  | E2 | 22.49 | 0.039282 |  |  |  | E2 | 17.82 |  |
|  |  | A1 | 24.2 | 0.016064 |  |  |  | A1 | 18.24 |  |
|  |  | A2 | 22.72 | 0.039282 |  |  |  | A2 | 18.05 |  |
|  | 8 | E1 | 22.95 | 0.021944 |  |  | 8 | E1 | 17.44 |  |
|  |  | E2 | 22.57 | 0.057912 |  |  |  | E2 | 18.46 |  |
|  |  | A1 | 25.06 | 0.012259 |  |  |  | A1 | 18.71 |  |
|  |  | A2 | 24.09 | 0.01468 |  |  |  | A2 | 18 |  |
|  | 9 | E1 | 22.97 | 0.027017 |  |  | 9 | E1 | 17.76 |  |
|  |  | E2 | 22.87 | 0.041235 |  |  |  | E2 | 18.27 |  |
|  |  | A1 | 24 | 0.010097 |  |  |  | A1 | 17.37 |  |
|  |  | A2 | 22.18 | 0.033493 |  |  |  | A2 | 17.28 |  |
|  | 10 | E1 | 24.48 | 0.012007 |  |  | 10 | E1 | 18.1 |  |
|  |  | E2 | 22.99 | 0.033493 |  |  |  | E2 | 18.09 |  |
|  |  | A1 | 23.81 | 0.014082 |  |  |  | A1 | 17.66 |  |
|  |  | A2 | 23.16 | 0.03125 |  |  |  | A2 | 18.16 |  |
|  |  |  |  |  |  |  |  |  |  |  |
