# Supplementary material for: Eutopic/ectopic endometrial apoptosis initiated by bilateral uterine artery occlusion: A new therapeutic mechanism for uterus-sparing surgery in adenomyosis
Source: PLoS One. 2017 Apr 13;12(4):e0175511. doi: 10.1371/journal.pone.0175511 (PMC5391022; doi:10.1371/journal.pone.0175511)
Supplement: S2 Table — (DOCX) [file pone.0175511.s002.docx]

| **Grey value**  **cases** | EuE | | | | | | | | |
| --- | --- | --- | --- | --- | --- | --- | --- | --- | --- |
|  |  | Bcl2 | caspase3 | Bax | TRADD | cyt-c | caspase4 | APF-1 | GAPDH |
| 1 | E1 | 17781.09 | 8898.562 | 5552.79 | 6107.619 | 2418.891 | 2425.234 | 9203.104 | 12050.38 |
|  | E2 | 9584.431 | 12226.36 | 5765.154 | 9869.912 | 7870.811 | 7074.497 | 7577.619 | 12245.81 |
| 2 | E1 | 11113.64 | 10667.4 | 3765.79 | 6776.497 | 6797.154 | 6087.983 | 5805.79 | 10392.23 |
|  | E2 | 9700.175 | 11569.28 | 4304.669 | 8683.276 | 7731.983 | 3492.033 | 5197.255 | 10105.86 |
| 3 | E1 | 3610.196 | 12734.45 | 1859.184 | 4416.205 | 7595.518 | 4051.012 | 6183.033 | 13094.74 |
|  | E2 | 12718.04 | 20350.09 | 4427.255 | 8594.669 | 10377.93 | 4056.205 | 6821.426 | 13006.55 |
| 4 | E1 | 8310.555 | 10072.6 | 3458.79 | 4339.912 | 5201.083 | 6534.154 | 10722.4 | 12522.79 |
|  | E2 | 8171.063 | 11024.6 | 3507.619 | 7726.255 | 11111.76 | 7050.761 | 11289.52 | 12198.21 |
| 5 | E1 | 13950.05 | 9842.518 | 3522.74 | 8575.569 | 7753.347 | 4465.983 | 6254.376 | 10665.81 |
|  | E2 | 12537.3 | 8716.518 | 4302.569 | 9489.447 | 6753.205 | 7402.711 | 5477.619 | 11449.43 |
| 6 | E1 | 14312.88 | 10008.15 | 1928.305 | 10066.69 | 7430.69 | 5815.205 | 5828.033 | 12283.18 |
|  | E2 | 8443.711 | 10215.57 | 3352.205 | 6807.933 | 6995.326 | 8740.347 | 10343.91 | 11430.28 |
| 7 | E1 | 8173.64 | 10046.74 | 3709.154 | 4071.033 | 6637.861 | 9547.912 | 10588.5 | 11053.1 |
|  | E2 | 8279.64 | 10353.45 | 4593.447 | 6726.69 | 8745.811 | 9190.104 | 9779.376 | 10634.18 |
| 8 | E1 | 10798.77 | 7146.919 | 1145.82 | 2379.941 | 3134.619 | 1780.477 | 7789.79 | 13374.28 |
|  | E2 | 4697.231 | 10345.49 | 4118.74 | 9565.619 | 4544.983 | 4562.347 | 10257.28 | 12603.98 |
| 9 | E1 | 3104.033 | 3161.619 | 1396.87 | 6879.205 | 6259.196 | 1971.477 | 7075.397 | 12855.88 |
|  | E2 | 2295.841 | 8010.832 | 6227.326 | 10248.74 | 6495.953 | 2558.326 | 8743.397 | 12533.23 |
| 10 | E1 | 7827.397 | 11534.08 | 3782.447 | 8534.497 | 7841.296 | 3130.376 | 6896.569 | 12936.59 |
|  | E2 | 1989.77 | 15150 | 3951.276 | 8150.447 | 11775.9 | 3856.719 | 5256.983 | 12442.52 |
|  |  |  |  |  |  |  |  |  |  |
|  |  |  |  |  |  |  |  |  |  |
| **Grey value**  **cases** | EuE | | | | | | | |  |
|  |  | GRP78 | Endo-G | caspase9 | CHOP | AIF | caspase8 | GAPDH |  |
| 1 | E1 | 8058.054 | 6778.518 | 5823.64 | 1151.255 | 3140.861 | 10647.1 | 13245.28 |  |
|  | E2 | 9218.619 | 5270.69 | 13727.05 | 11298.64 | 7586.69 | 8148.64 | 12253.96 |  |
| 2 | E1 | 11136.45 | 3098.548 | 5948.74 | 6233.276 | 1099.355 | 971.062 | 10019.5 |  |
|  | E2 | 10833.81 | 14822.98 | 7876.326 | 7303.69 | 4487.225 | 7328.397 | 10898.03 |  |
| 3 | E1 | 12272.57 | 2335.134 | 8461.347 | 8506.104 | 639.355 | 7105.811 | 11812.35 |  |
|  | E2 | 13323.35 | 3760.983 | 10115.47 | 11818.79 | 2422.154 | 9565.054 | 11497.18 |  |
| 4 | E1 | 14729.23 | 7157.912 | 8535.225 | 590.456 | 4828.468 | 11475.64 | 12588.96 |  |
|  | E2 | 14135.54 | 9057.69 | 9817.246 | 2970.326 | 3722.861 | 11464.23 | 13166.45 |  |
| 5 | E1 | 10357.86 | 5591.033 | 8033.69 | 4386.518 | 6195.468 | 5435.811 | 11646.69 |  |
|  | E2 | 7280.225 | 9418.861 | 8446.397 | 7084.882 | 11587.28 | 9883.69 | 11008.18 |  |
| 6 | E1 | 9139.589 | 8450.104 | 6966.347 | 7841.225 | 2565.64 | 2192.033 | 11121.33 |  |
|  | E2 | 8032.083 | 12787.98 | 6521.983 | 1890.669 | 2336.861 | 1717.255 | 11513.21 |  |
| 7 | E1 | 10155.84 | 4211.497 | 4375.983 | 6668.326 | 3806.104 | 756.042 | 11368.55 |  |
|  | E2 | 9574.811 | 8269.225 | 7108.397 | 13062.35 | 11243.03 | 8877.154 | 10391.38 |  |
| 8 | E1 | 14618.4 | 4511.305 | 4388.154 | 1754.619 | 1434.376 | 7721.418 | 11495.86 |  |
|  | E2 | 14797 | 10047.52 | 8233.004 | 1420.79 | 9350.326 | 10042.64 | 12038.45 |  |
| 9 | E1 | 8006.69 | 1773.255 | 4560.368 | 5592.882 | 1996.477 | 2350.397 | 12723.1 |  |
|  | E2 | 9721.79 | 4254.912 | 3635.811 | 6889.004 | 10377.47 | 11533.35 | 12597.93 |  |
| 10 | E1 | 10023.76 | 986.284 | 5609.589 | 7535.489 | 8246.154 | 8850.418 | 12939.76 |  |
|  | E2 | 10491.74 | 9130.125 | 2850.962 | 10934.44 | 7372.024 | 8903.66 | 11952.3 |  |
|  |  |  |  |  |  |  |  |  |  |
|  |  |  |  |  |  |  |  |  |  |
| **Grey value** | EE | | | | | | | | |
| **cases** |  | Bcl2 | caspase3 | Bax | TRADD | cyt-c | caspase4 | APF-1 | GAPDH |
| 1 | A1 | 7740.363 | 6558.579 | 5238.276 | 8190.154 | 7406.225 | 4194.79 | 7455.861 | 12309.98 |
|  | A2 | 10075.41 | 8659.878 | 10257.64 | 10145.57 | 5179.104 | 4480.134 | 8462.083 | 12179.52 |
| 2 | A1 | 9198.64 | 6756.205 | 2554.82 | 3648.912 | 6112.79 | 4986.912 | 5188.426 | 10321.03 |
|  | A2 | 6382.397 | 8039.012 | 2788.062 | 7382.518 | 7300.74 | 3097.154 | 8404.569 | 11690.03 |
| 3 | A1 | 10076.81 | 7430.164 | 6310.74 | 7758.205 | 714.577 | 4074.548 | 9070.619 | 11336.52 |
|  | A2 | 9612.59 | 9439.2 | 5986.569 | 6974.79 | 2148.841 | 5697.74 | 9132.74 | 10838.03 |
| 4 | A1 | 7536.448 | 6492.656 | 5359.64 | 5715.79 | 4203.154 | 5630.962 | 9937.083 | 12338.5 |
|  | A2 | 8695.42 | 8897.697 | 8408.397 | 8775.154 | 5400.397 | 6302.962 | 9266.518 | 11612.3 |
| 5 | A1 | 7058.154 | 7940.518 | 2168.376 | 1947.305 | 7740.761 | 2085.548 | 5834.79 | 11132.86 |
|  | A2 | 6810.225 | 7992.154 | 4060.083 | 6850.175 | 6396.397 | 5890.719 | 5724.326 | 10772.33 |
| 6 | A1 | 6778.154 | 7484.104 | 1938.012 | 4886.033 | 8580.104 | 5179.619 | 7465.841 | 12102.69 |
|  | A2 | 5492.154 | 9062.104 | 5094.669 | 8622.347 | 10585.1 | 6352.397 | 7377.962 | 10876.72 |
| 7 | A1 | 5267.811 | 7059.033 | 3359.376 | 1772.305 | 11452.03 | 5367.548 | 4545.77 | 10918.67 |
|  | A2 | 6517.397 | 8472.569 | 4901.962 | 1799.184 | 10529.52 | 6942.79 | 7435.376 | 12327.26 |
| 8 | A1 | 10413.99 | 9514.422 | 7516.518 | 9986.255 | 803.284 | 3523.376 | 9468.69 | 11199.81 |
|  | A2 | 13127.05 | 7517.074 | 8205.518 | 9595.255 | 7165.912 | 5599.347 | 9536.74 | 11777.81 |
| 9 | A1 | 13373.2 | 4778.246 | 3095.861 | 9551.861 | 14633.35 | 4877.69 | 8335.054 | 10345.93 |
|  | A2 | 9874.64 | 13925.88 | 5034.518 | 10332.45 | 11625.15 | 5953.589 | 9270.468 | 11245.13 |
| 10 | A1 | 9035.317 | 8160.933 | 1996.841 | 5870.841 | 8865.296 | 5446.66 | 7753.761 | 10606.81 |
|  | A2 | 3711.962 | 14475.78 | 7052.468 | 8811.79 | 11374.88 | 6744.832 | 7284.589 | 11894.23 |
|  |  |  |  |  |  |  |  |  |  |
|  |  |  |  |  |  |  |  |  |  |
| **Grey value** | EE | | | | | | | |  |
| **cases** |  | GRP78 | Endo-G | caspase9 | CHOP | AIF | caspase8 | GAPDH |  |
| 1 | A1 | 10720.64 | 2742.669 | 801.042 | 1196.82 | 2077.669 | 1158.577 | 12365.5 |  |
|  | A2 | 8208.104 | 10785.98 | 3126.69 | 7396.64 | 6523.69 | 4916.518 | 11603.28 |  |
| 2 | A1 | 7723.619 | 2614.619 | 2077.598 | 3602.397 | 7929.225 | 6458.397 | 10863.81 |  |
|  | A2 | 6427.012 | 3351.861 | 2644.669 | 1805.033 | 6878.083 | 8696.69 | 10280.26 |  |
| 3 | A1 | 6799.962 | 13911.3 | 2394.083 | 8432.397 | 762.648 | 1156.406 | 12017.88 |  |
|  | A2 | 7327.184 | 16201.54 | 2545.376 | 15229 | 2903.983 | 2196.447 | 11906.93 |  |
| 4 | A1 | 7329.154 | 5754.983 | 3620.962 | 3869.861 | 1151.305 | 319.728 | 12210.18 |  |
|  | A2 | 5540.669 | 6483.347 | 3696.74 | 7724.518 | 10078.1 | 4517.397 | 10977.69 |  |
| 5 | A1 | 6653.841 | 8204.296 | 780.577 | 7736.811 | 563.87 | 1523.912 | 11853.38 |  |
|  | A2 | 7649.083 | 4862.054 | 640.042 | 6295.882 | 3788.912 | 7029.69 | 12451.79 |  |
| 6 | A1 | 6467.548 | 2454.447 | 3655.376 | 10066.88 | 536.678 | 1088.891 | 12963.62 |  |
|  | A2 | 8497.962 | 8143.468 | 4208.305 | 10406.93 | 8940.225 | 9827.497 | 12662.01 |  |
| 7 | A1 | 7789.669 | 7038.468 | 1421.991 | 7393.983 | 1936.861 | 3251.861 | 12531.38 |  |
|  | A2 | 8290.184 | 10770.05 | 3886.548 | 7466.497 | 7382.054 | 7502.497 | 12060.84 |  |
| 8 | A1 | 7789.669 | 8292.882 | 719.627 | 432.678 | 3454.154 | 6723.033 | 10731.64 |  |
|  | A2 | 7330.134 | 5877.225 | 2132.548 | 2960.326 | 8552.326 | 8930.497 | 13898.18 |  |
| 9 | A1 | 9924.841 | 9032.004 | 9775.953 | 9138.196 | 1902.376 | 1316.184 | 10888.28 |  |
|  | A2 | 11062.85 | 12904.61 | 4018.347 | 6102.054 | 12887.73 | 12089.15 | 10688.69 |  |
| 10 | A1 | 8850.196 | 2463.154 | 5265.196 | 5311.853 | 14050.86 | 10816.47 | 11064.47 |  |
|  | A2 | 11351.3 | 1546.062 | 7187.175 | 14401.22 | 5602.388 | 11687.08 | 11202.76 |  |
|  |  |  |  |  |  |  |  |  |  |
