# Supplementary material for: Eutopic/ectopic endometrial apoptosis initiated by bilateral uterine artery occlusion: A new therapeutic mechanism for uterus-sparing surgery in adenomyosis
Source: PLoS One. 2017 Apr 13;12(4):e0175511. doi: 10.1371/journal.pone.0175511 (PMC5391022; doi:10.1371/journal.pone.0175511)
Supplement: S3 Table — (DOCX) [file pone.0175511.s003.docx]

| **Apoptosis-related factors** | **Hour of hypoxia**  **Primary cells**  **Grey value** | 0 | 6 | 12 | 24 | 48 |
| --- | --- | --- | --- | --- | --- | --- |
| caspase3 | CE | 3021.65 | 5601.69 | 6585.37 | 9273.3 | 10720.5 |
|  |  | 4429.59 | 6322.68 | 12355.3 | 11496 | 14108.2 |
|  |  | 5033.91 | 6906.23 | 5408.76 | 10451.5 | 12006 |
|  | EE | 1698.18 | 2786.98 | 4158.23 | 10805.4 | 13274 |
|  |  | 1238.28 | 3651.35 | 5661.93 | 13587.3 | 13310 |
|  |  | 1055.6 | 1568.21 | 4942.18 | 11133.3 | 15742.1 |
|  | EuE | 2095.65 | 2735.21 | 5697.81 | 6553.66 | 12317.7 |
|  |  | 3051.57 | 5871.59 | 7897.49 | 8764.34 | 11713.2 |
|  |  | 2164.26 | 4161.1 | 6113.1 | 6522.13 | 11502.4 |
| Bcl2 | CE | 5557.97 | 4987.42 | 4801.42 | 1971.98 | 1319.79 |
|  |  | 9538.47 | 7363.93 | 7119.64 | 3944.91 | 1699.65 |
|  |  | 9721.25 | 8507.05 | 5841.15 | 3499.96 | 1242.04 |
|  | EE | 11151.9 | 6862.2 | 5878.47 | 3839.91 | 1783.06 |
|  |  | 19163 | 7351.97 | 8515.9 | 6792.71 | 2126.15 |
|  |  | 10803.7 | 4189.15 | 3721.93 | 3324.13 | 1173.82 |
|  | EuE | 9706.15 | 3116.28 | 3098.76 | 2913.83 | 2426.55 |
|  |  | 8071.39 | 7562.17 | 4864.4 | 3573.23 | 1404.6 |
|  |  | 9404.95 | 10365.3 | 4983.83 | 4508.59 | 1666.79 |
| Endo-G | CE | 7979.2 | 10476.5 | 9965.27 | 10071.5 | 11676 |
|  |  | 7915.85 | 11283.9 | 10542.1 | 11572.8 | 13416.4 |
|  |  | 8015.61 | 9061.61 | 9704.39 | 9467.32 | 14738.7 |
|  | EE | 989.113 | 2239.01 | 5179.35 | 6554.64 | 8622.1 |
|  |  | 1962.06 | 2258.38 | 5625.88 | 7103.3 | 15472.8 |
|  |  | 1134.82 | 3050.15 | 7993.56 | 11105.4 | 16390.8 |
|  | EuE | 8752.02 | 8333.95 | 18060.3 | 15787 | 20514 |
|  |  | 3807.4 | 6021.92 | 13721.8 | 15478 | 16036.2 |
|  |  | 1970.6 | 5301.35 | 9099.93 | 12433.9 | 11702.7 |
| caspase8 | CE | 5665.78 | 8376.56 | 10003.9 | 10143.5 | 11099.2 |
|  |  | 8106.83 | 10264.5 | 12333 | 13802.7 | 14096.1 |
|  |  | 4379.59 | 8209.22 | 12054.8 | 14059.6 | 13078.4 |
|  | EE | 2048.26 | 3620.08 | 3680.52 | 6152.23 | 12821.5 |
|  |  | 1936.28 | 3639.13 | 5834.37 | 11583 | 14111.2 |
|  |  | 3723.73 | 3994.59 | 9800.6 | 7925.29 | 13764.6 |
|  | EuE | 1664.36 | 6527.47 | 7465.05 | 8316.85 | 10634 |
|  |  | 3631 | 4445.25 | 7799.37 | 10008.2 | 11274.5 |
|  |  | 7331.13 | 7684.71 | 8579.83 | 10967.5 | 16262.8 |
| GRP78 | CE | 13780.3 | 16579.5 | 17058.8 | 19026.4 | 20161.3 |
|  |  | 11887.4 | 14638.3 | 13723.2 | 15839.9 | 17870.3 |
|  |  | 17107.7 | 16301.3 | 18449.4 | 19551.1 | 20157.8 |
|  | EE | 1828.99 | 4021.35 | 12462.8 | 18326.4 | 23249.9 |
|  |  | 9870.44 | 11674.7 | 10463.3 | 11817.8 | 16919.4 |
|  |  | 5881.64 | 9586.95 | 9672.71 | 13457.7 | 18472.5 |
|  | EuE | 5894.56 | 12717.7 | 15120.7 | 14583.7 | 17311 |
|  |  | 10929.3 | 12379.1 | 14107.8 | 17274.2 | 15884.4 |
|  |  | 11696.1 | 10762.5 | 15029.4 | 16354.8 | 18954 |
| Bax | CE | 9424.58 | 13866.7 | 16206.6 | 22410.4 | 22463.3 |
|  |  | 7802.32 | 10814.1 | 11493.6 | 13186.2 | 16768.4 |
|  |  | 5757.54 | 8260.1 | 8872.44 | 13044.9 | 16492.5 |
|  | EE | 5324.49 | 8856.08 | 9068.4 | 11263.4 | 15473.8 |
|  |  | 2417.38 | 4917.93 | 6319.25 | 8121.61 | 9524.85 |
|  |  | 3765.55 | 5621.03 | 6688.5 | 7871.91 | 13782.7 |
|  | EuE | 4220.69 | 5374.81 | 8075.37 | 8114.88 | 8180.42 |
|  |  | 2721.21 | 3813.5 | 4969.42 | 9923.15 | 11221 |
|  |  | 2712.69 | 7613.81 | 7593.91 | 11643 | 11784.9 |
| GAPDH | CE | 24388.1 | 24735.2 | 25096.2 | 23886.2 | 24314.7 |
|  |  | 24468.6 | 24724.6 | 25556.3 | 24122.5 | 23717.1 |
|  |  | 20990.9 | 22474.8 | 21510.5 | 21513.3 | 21961.6 |
|  | EE | 25513.5 | 25680.1 | 25130.4 | 25715.5 | 25372 |
|  |  | 24145.3 | 25053.3 | 24903.6 | 25417.6 | 23617.1 |
|  |  | 21138.1 | 22128.4 | 22854.5 | 22006.2 | 20480.2 |
|  | EuE | 24616.7 | 23895.3 | 24025.8 | 23018.4 | 23791.9 |
|  |  | 24596.7 | 24361.4 | 24767.5 | 24712.1 | 25329.6 |
|  |  | 23217.2 | 24192.8 | 24177.6 | 23931.5 | 23841.9 |
|  |  |  |  |  |  |  |
